# Insecticidal Activity of *Trichilia hirta* L. (Sapindales: Meliaceae) Extracts Against *Spodoptera frugiperda* (Lepidoptera: Noctuidae) Larvae and the Identification of Bioactive Compounds

**DOI:** 10.1002/cbdv.202503155

**Published:** 2026-03-25

**Authors:** Mayara Barreto de Souza Arantes, Renata Rodrigues da Silva Robaina, Thalya Soares Ribeiro Nogueira, João Victor Panisset Lima Barcelos, Wanderson Rosa da Silva, Guilherme Ferreira Soares Passos, Renata Cunha Pereira, Ludimila Simões Peçanha, Gabriel Garreto dos Santos, Lucas Silva Abreu, Ivo José Curcino Vieira, Raimundo Braz Filho, Richard Ian Samuels, Gerson Adriano Silva

**Affiliations:** ^1^ Laboratory of Entomology and Plant Pathology, Center for Agricultural Sciences and Technology Universidade Estadual do Norte Fluminense Darcy Ribeiro UENF Campos dos Goytacazes Rio de Janeiro Brazil; ^2^ Chemical Sciences Laboratory, Science and Technology Center Universidade Estadual do Norte Fluminense Darcy Ribeiro UENF Campos dos Goytacazes Rio de Janeiro Brazil; ^3^ Institute of Chemistry Universidade Federal Fluminense, UFF Niterói Rio de Janeiro Brazil

**Keywords:** bioactive compounds, larval toxicity, limonoids, natural products, plant extracts

## Abstract

Studying plants with insecticidal activity and the compounds responsible for this type of activity is a promising approach for discovering new insecticides. Plants from the Meliaceae family are rich in limonoids, compounds with proven insecticidal action against certain pests. This study investigated the insecticidal activity of the crude methanolic extracts from *Trichilia hirta* fruits against *Spodoptera frugiperda* larvae. An initial toxicity bioassay showed 100% larval mortality when testing *T. hirta* extract. The crude extract was partitioned using ethyl acetate, dichloromethane, and n‐butanol, and the LD_50_ dose (785.244 mg kg^−^^1^) was used to test the solvent fractions against the larvae. The ethyl acetate fraction caused the highest mortality and reduced larval weight gain. This fraction was subjected to column chromatography, yielding 12 fractions, of which, fractions 5 to 9 inhibited larval weight gain and caused mortality. LC‐ESI‐HRMS/MS analysis of the ethyl acetate fraction enabled the identification of four compounds: (1) noreugenin, (2) methyl 6,11*β*‐dihydroxy‐12*α*‐(2‐methylpropanoyloxy‐3,7‐dioxo‐14*β*, 15*β*‐epoxy‐1,5‐meliacadien‐29‐oate, (3) hirtinol A, and (4) 5α‐androstane‐3*β*,17*β*‐diol. This is the first study to report the presence of noreugenin in this plant genus. Future studies will be carried out to isolate other substances with promising insecticidal activities.

## Graphical abstract

Fruits of *Trichilia hirta* were extracted and fractionated according to solvent polarity. The resulting partitions were screened for insecticidal activity by evaluating larval mortality and weight after 7 and 14 days. LC‐MS analysis of the most active fraction enabled the annotation of secondary metabolites potentially associated with the observed bioactivity.

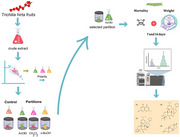

## 1 Introduction

Plants produce a wide range of bioactive secondary metabolites, which can serve as model molecules for the development of new insecticides [1, 2]. Research into compounds of botanical origin has gained momentum over the last four decades. The primary goal of this type of research is to identify novel compounds that could be used as alternatives to conventional (organosynthetic) pesticides, thereby reducing the utilization of products that are harmful to the environment and human health [3]. Notable examples of botanical pesticides include azadirachtin from the plant *Azadirachta indica* (Meliaceae), pyrethroids derived from pyrethrin, originally isolated from *Chrysanthemum cinerariaefolium* (Asteraceae), and neonicotinoids based on nicotine, which are found in species of the genus *Nicotiana* (Solanaceae) [4, 5, 6, 7].

South America is home to approximately 120 000 plant species, with Brazil hosting around 30% of this biodiversity []. Brazilian biomes contain many plants with insecticidal effects, making them valuable sources of compounds for pest control [9]. The Meliaceae family comprises approximately 59 genera and 745 plant species; six of these genera are native to Brazil (*Cedrela*, *Cabralea*, *Swietenia*, *Carapa*, *Guarea*, and *Trichilia*) [8, 10, 11]. Species of the genus *Trichilia* produce a wide variety of compounds that exhibit several biological activities, including antimicrobial, anti‐inflammatory, antioxidant, insecticidal, repellent, and antifeedant properties in insects [12, 13].

The biological activity of Meliaceae is mainly associated with the presence of limonoids, highly oxygenated modified triterpenoids. Studies on limonoids from different species of Meliaceae of the genus *Khaya* highlighted their potential in the development of therapeutic agents, including anticancer, antimalarial, hepatoprotective, anti‐inflammatory, neuroprotective, antimicrobial, and antifungal properties [14]. Limonoids also exhibit insecticidal activity against larvae of the cotton leafworm *Spodoptera littoralis* (Lepidoptera: Noctuidae), adult *Rhyzopertha dominica* (Coleoptera: Bostrychidae), and *Tribolium castaneum* (Coleoptera: Tenebrionidae). Extracts from the neem tree, *Azadirachta indica*, have been extensively investigated for their potential to control insect pests [15, 16, 17]. Azadirachtin was the first limonoid to be isolated from *A. indica* [18, 19]. Neem continues to be widely used for pest control and has demonstrated high levels of insecticidal activity [20]. Azadirachtin is currently used as the active ingredient in various commercial insecticides in Brazil [21].

A bibliographic survey of compounds isolated from plants of the genus *Trichilia* indicated that most are terpenoids [22]. Based on this survey, 227 limonoids were identified from 21 species of *Trichilia* between 1966 and 2020 [23]. During the period from 2013 to 2023, around 305 new compounds were isolated and identified from *Trichilia* species, among which limonoids were predominant, representing 61% of the total [24]. The insecticidal activity of fruit extracts from *Trichilia elegans* and *Trichilia catiguá* was tested against *S. frugiperda* larvae, and limonoids, coumarins, and steroids were identified. The toxicity assays showed that the hexane and methanolic extracts of *T. elegans* delayed larval development and inhibited molting, resulting in 100% larval mortality. However, *T. catiguá* extracts caused approximately 50% mortality but did not interfere with the molting process [25, 26]. In *Trichilia americana*, the ethyl acetate and acetone partitions caused 90% and 50% larval mortalities in *Copitarsia decolora* (Lepidoptera: Noctuidae), respectively. Sublethal effects were also observed, including reduced larval weight, prolonged larval duration, malformed pupae, and reduced fertility of adults derived from treated larvae [27]. In contrast, aqueous and non‐aqueous extracts of *Trichilia pallida* showed low levels of toxicity to *Tuta absoluta* (Lepidoptera: Gelechiidae) larvae, causing mortalities of 13.2% and 7.7%, respectively [28].

Thus, the study of *Trichilia* species is important because they may contain compounds similar to those found in *Melia* and *Azadirachta*, and several studies have demonstrated promising insecticidal activity in *Trichilia* species [29]. Despite evidence of insecticidal activity within the genus, *Trichilia hirta* remains poorly studied with regard to both its extracts and isolated compounds. Only two studies addressing its insecticidal activity have been found in scientific databases [30, 31]. The first study tested extracts of *T. hirta* leaves, bark, and wood against *Peridroma saucia* (Lepidoptera: Noctuidae) and *Spodoptera litura* (Lepidoptera: Noctuidae) and found the wood extract to be the most active, causing 100% mortality within 7 days [30]. The second study evaluated ethanolic extracts from different plant parts of *T. hirta* and *Tabernaemontana cymosa* against third‐ and fourth‐instar *Aedes aegypti* (Diptera: Culicidae) larvae. *Trichilia hirta* bark extract showed no larvicidal activity, whereas the seed extract showed moderate larvicidal activity (LC_50_ = 219.22 mg L^−^^1^), particularly when compared with *T. cymosa* seed extracts (LC_50_ = 35.1 mg L^−^^1^) [31]. Although the phytochemical profile of *T. hirta* has been elucidated, its insecticidal potential remains underexplored. The chemical profile shows that the extracts are primarily composed of limonoids [22]; however, specific limonoids present in *T. hirta* have not yet been investigated for their efficacy against different pests. Further research on this species could uncover compounds with potential for the development of new pesticides for agricultural use.

The fall armyworm, *Spodoptera frugiperda* (J. E. Smith) (Lepidoptera: Noctuidae), is a polyphagous pest responsible for significant yield losses in crops such as rice, wheat, corn, sorghum, soybeans, cotton, vegetables, and fruit crops [32, 33]. Reports indicate that *S. frugiperda* feeds on more than 350 host plants, and this pest is capable of attacking approximately 76 plant families [32]. Originally native to tropical and subtropical America, this species has spread globally and is now considered a major pest in Europe, Africa, Asia, and Oceania [33, 34, 35, 36]. Chemical control and the use of resistant cultivars are the primary methods currently used for managing *S. frugiperda*. However, the widespread and intensive use of insecticides has led to the development of resistance, increasing the reliance on more toxic and environmentally harmful chemicals [36]. This pest causes substantial losses to growers by reducing crop productivity and generating economic damage to important crops such as maize, cotton, and sorghum [37]. Resistance of *S. frugiperda* to insecticides has been reported since 1976, and current data shows more than 200 cases of resistance to chemical insecticides and to Cry proteins expressed in Bt crops [38, 39].

Considering the global spread of this pest and the insecticidal potential of compounds found in species of the family Meliaceae, it is important to investigate whether metabolites from *T. hirta* exhibit activity against *S. frugiperda*. The findings here highlight the insecticidal potential of *T. hirta*; however, despite these promising early studies, the species remains largely unexplored, and only a limited number of studies are available in the literature addressing its insecticidal potential. Moreover, although *T. hirta* has a known chemical profile, little information exists regarding the insecticidal properties of its individual compounds. Therefore, the main objective of this study was to identify secondary metabolites from *T. hirta* (Meliaceae) and to evaluate their insecticidal activity against *S. frugiperda*.

## 2 Results

### 2.1 Insecticidal Activity of the *Trichilia hirta* Methanolic Extract

The *T. hirta* fruit methanolic extract caused 68% mortality after 7 days (*F*_1,10_ = 88.95; *p* < 0.001) and affected the weight (*F*_1,10_ = 5.41; *p* < 0.001) of the surviving *S. frugiperda* larvae. Fourteen days after the start of the bioassay, 100% mortality was observed in *S. frugiperda* larvae exposed to *T. hirta* extracts (Table 1). The median lethal dose (LD_50_) was 785.2 mg of extract per kg of diet. The estimated LD_90_ was 2594.7 mg of extract per kg of diet (Table 2).

### 2.2 Bioactivity of the Partitions of *Trichilia hirta* Extracts

**FIGURE 1:**
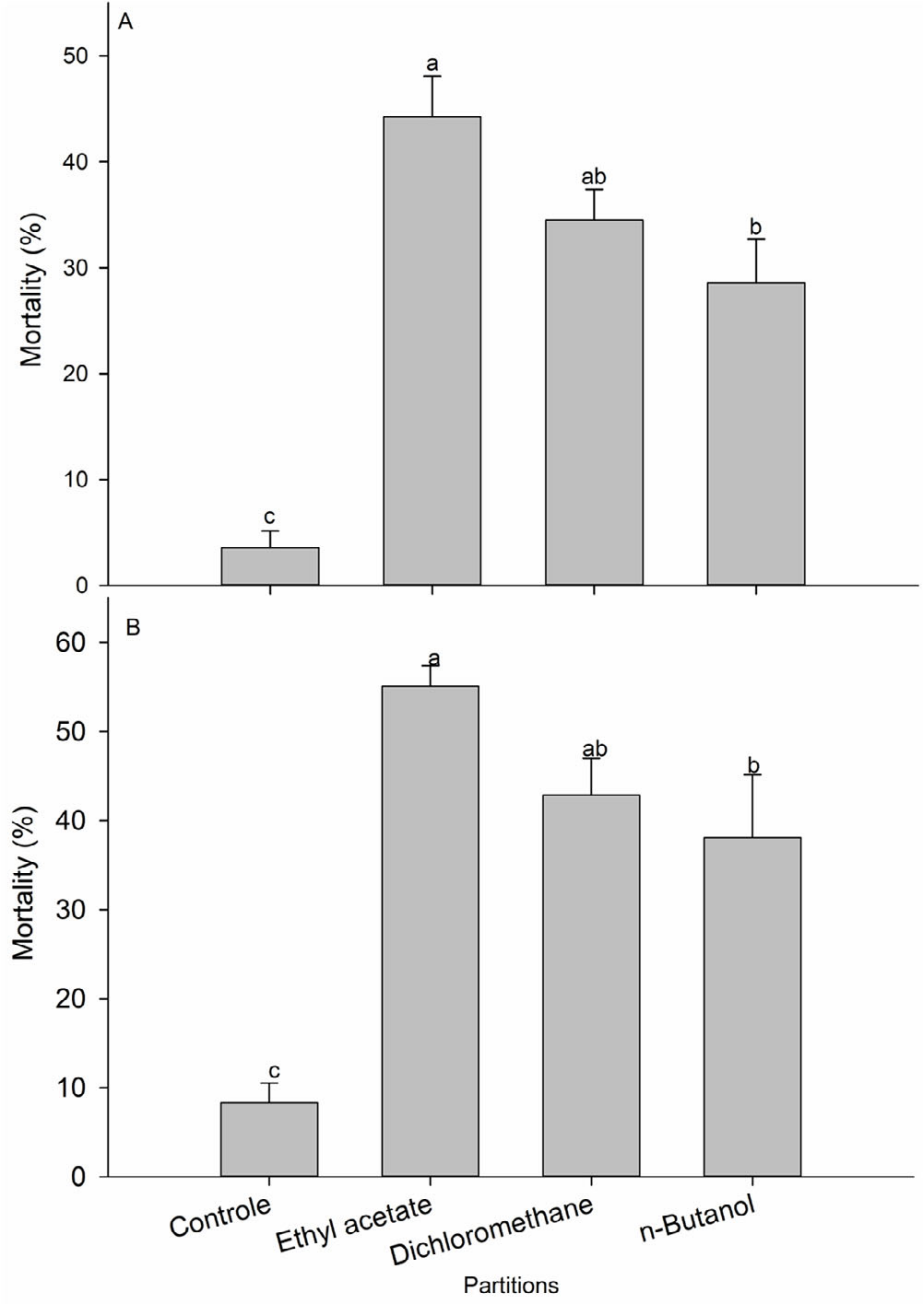
Mortality (means ± standard errors) of *Spodoptera frugiperda* larvae after exposure to *Trichilia hirta* extract partitions after 7 days (A) and 14 days (B) (*n* = 336). Means followed by different letters were significantly different from the control group according to Tukey's test (*p* < 0.05).

**FIGURE 2:**
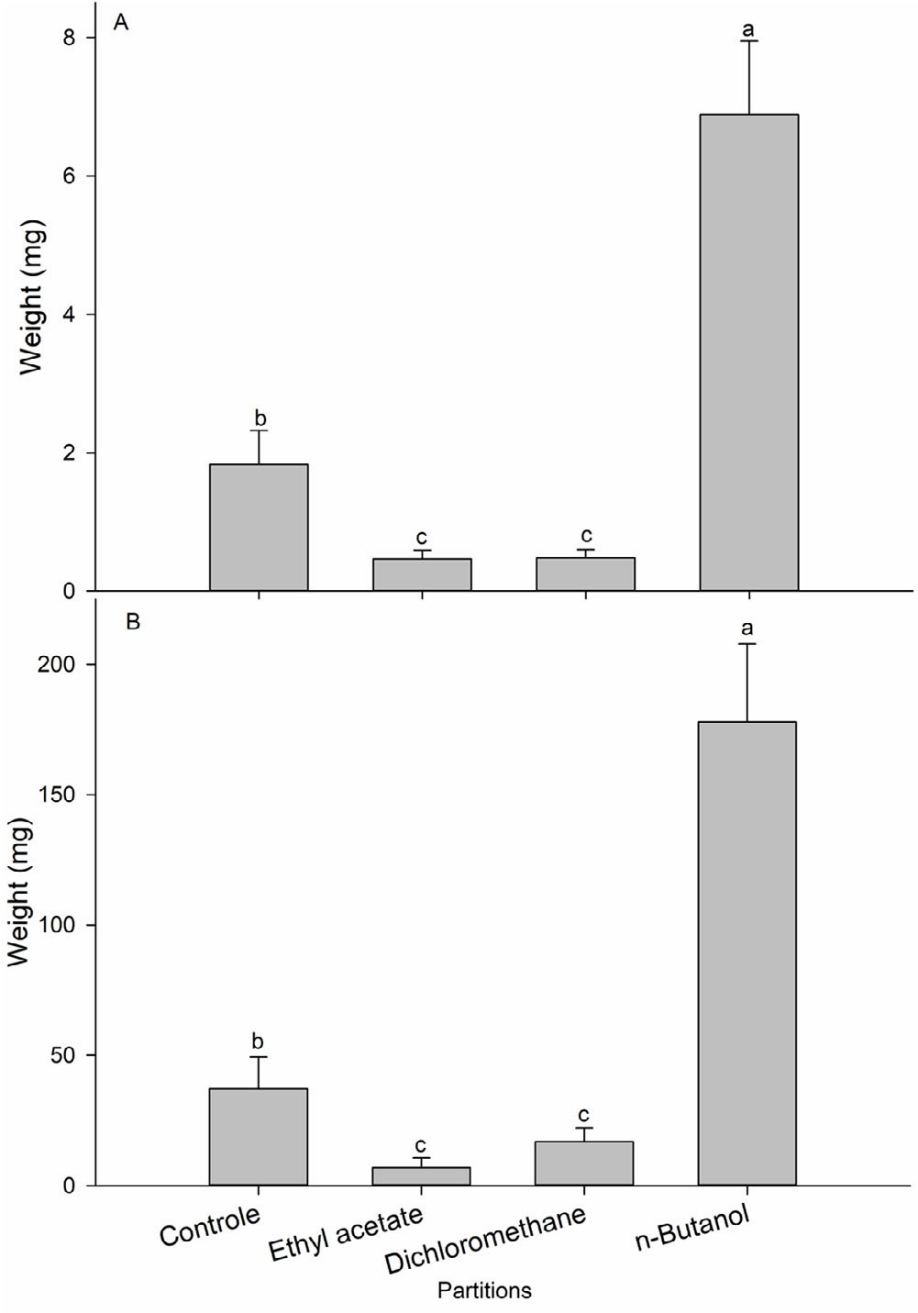
Fresh weight (means ± standard errors) of *Spodoptera frugiperda* larvae following exposure to *Trichilia hirta* partitions after 7 days (A) and 14 days (B) (*n* = 336). Means followed by different letters were significantly different from the control group according Tukey's test (*p* < 0.05).

The LD_50_ for *T. hirta* methanolic extracts (785.244 mg kg^−1^) was used as the reference dose to test the insecticidal action of ethyl acetate, dichloromethane, and *n*‐butanol partitions against *S. frugiperda* larvae. There were significant differences in larval mortality after 7 days (*F*_3,20_ = 19.77; *p* < 0.001) and 14 days (*F*_3,20_ = 17.39; *p* < 0.001) exposure to the *T. hirta* methanolic partition extracts. After 7 days, the highest mortality occurred in larvae exposed to the ethyl acetate partition, followed by dichloromethane and *n*‐butanol partitions. After 14 days of exposure, there was an increase in larval mortality for all partitions tested here. The mortalities observed at seven and 14 days differed statistically from the control group mortality (< 10%) (Figure 1). The larvae that survived a 7 day exposure to the diet containing the treatments (*F*_3,20_ = 160.59; *p* < 0.001) and a 14 day exposure (*F*_3,20_ = 140.94; *p* < 0.001) showed modifications in biomass accumulation. After 7 days, the mean weight of the larvae in the control group was 1.82 mg, which was higher than the weight of the larvae exposed to dichloromethane and ethyl acetate (0.48 and 0.46 mg, respectively). Among the larvae that survived a 14‐day exposure to the partitions, the ethyl acetate (7.02 mg) and dichloromethane (16.89 mg) partitions caused the greatest reduction in biomass accumulation, showing a marked decrease in larval weight compared with the control group, which reached a mean weight of 37.2 mg (Figure 2).

### 2.3 Biological Activity of Fractions From *Trichilia hirta* Ethyl Acetate (AcOEt) Partitions

The ethyl acetate fraction was chosen for further testing due to the high mortality rate of *S. frugiperda* following ingestion of this fraction and its ability to reduce larval weight gain (Figures 1 and 2). The partitioning of the ethyl acetate fraction from *T. hirta* resulted in 12 fractions. To test the insecticidal activity, a dose corresponding to 1/5 of the LD_50_ value of the crude extract (157.0 mg per kg of diet, based on the LD_50_ of 785.244 mg per kg) was used. There were significant differences in *S. frugiperda* larval mortality after a 7‐day (*F*_12,26_ = 29.324; *p* < 0.001) and 14‐day (*F*_12,26_ = 35.753; *p* < 0.001) exposure to the fractions.

**FIGURE 3:**
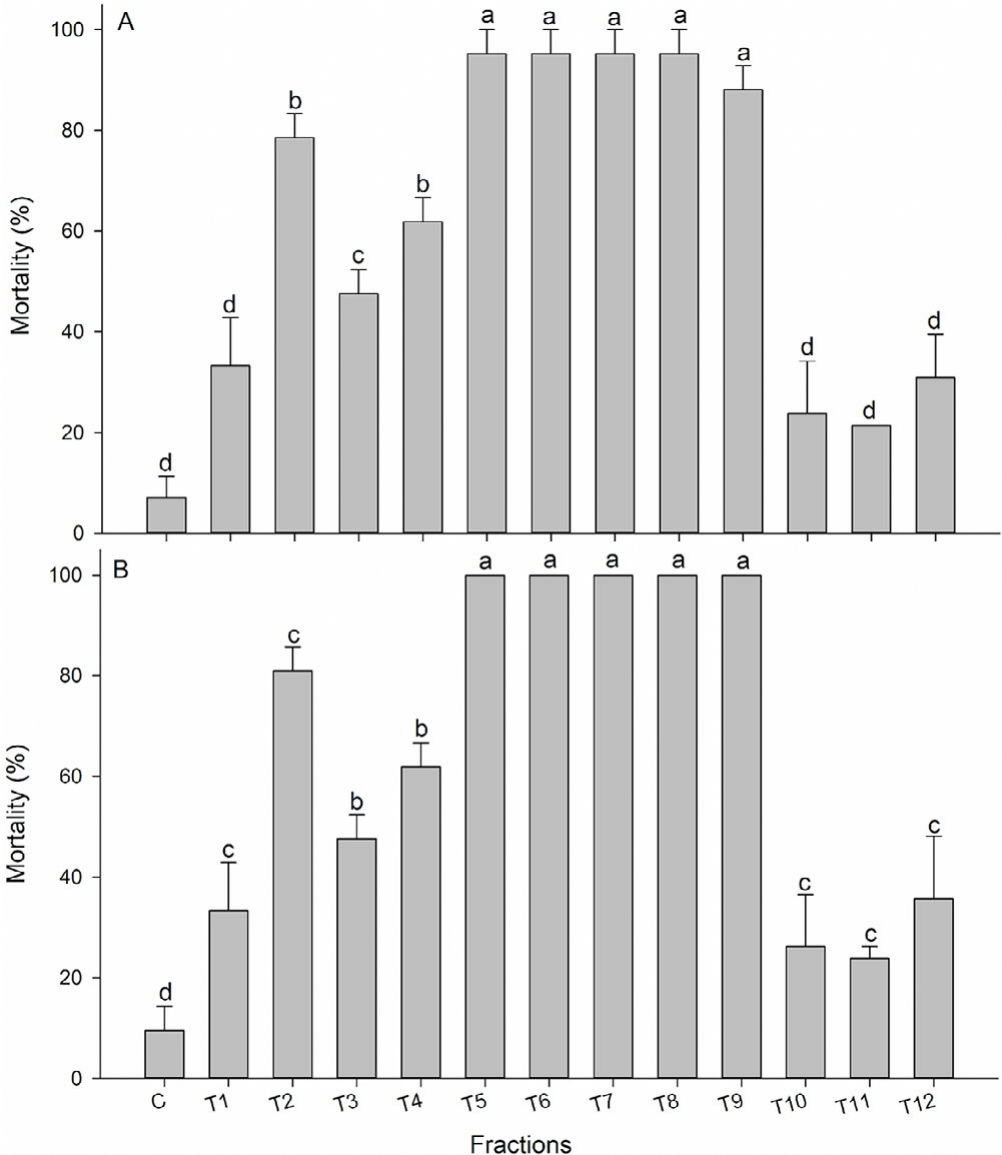
Mortality (means ± standard errors) of *Spodoptera frugiperda* larvae after exposure to fractions obtained from *Trichilia hirta* extract ethyl acetate partitions at 7 days (A) and 14 days (B) (*n* = 1092). Means followed by different letters were significantly different when compared to the control group according to the Scott‐Knott test (*p* < 0.05).

**FIGURE 4:**
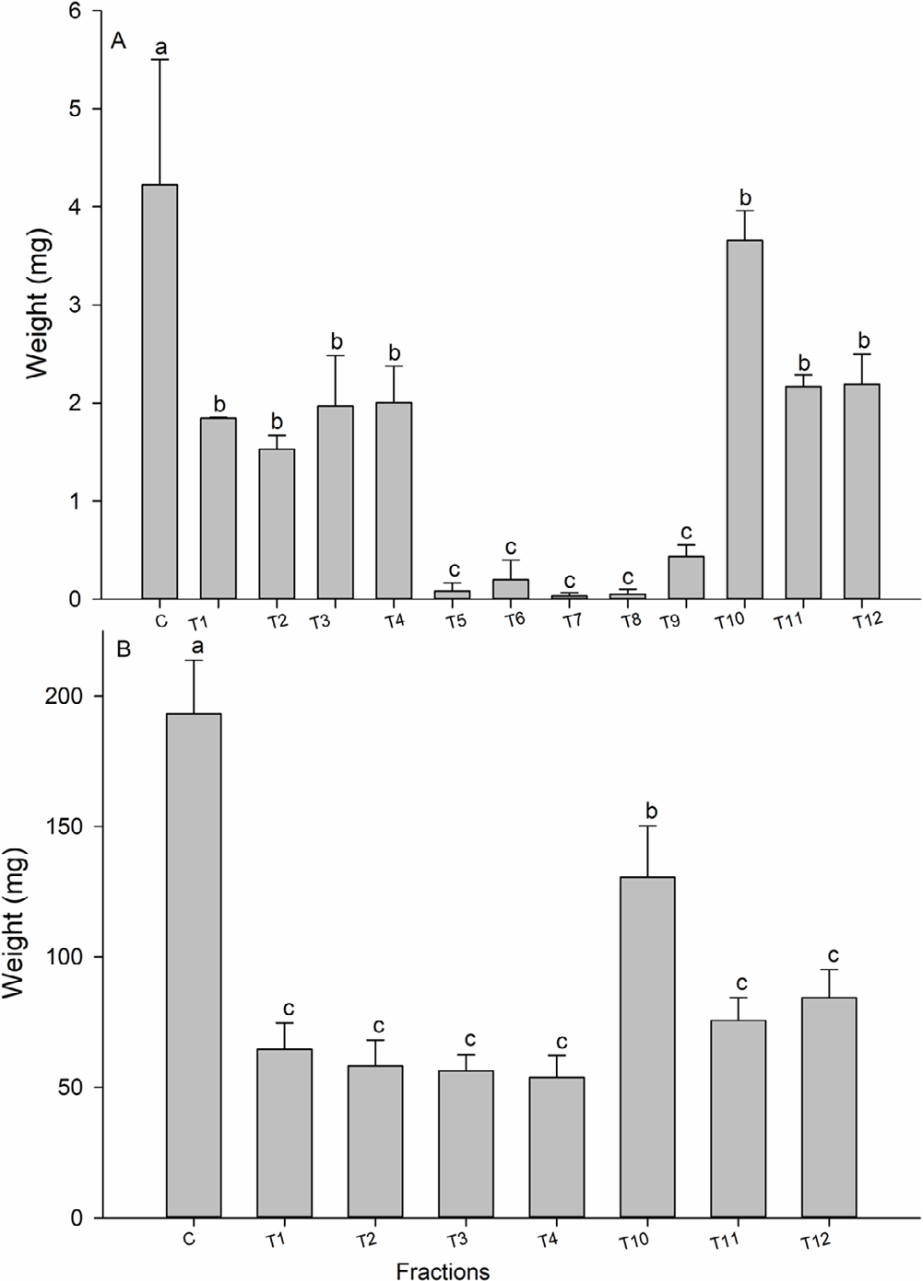
Fresh weight (means ± standard errors) of *Spodoptera frugiperda* larvae after exposure to fractions obtained from *Trichilia hirta* extract ethyl acetate partitions after 7 days (A) and 14 days (B) (*n* = 1092). Means followed by different letters were significantly different when compared to the control group according to the Scott‐Knott test (*p* < 0.05).

On the 7th day of evaluation, only fractions 10 and 12 did not differ from the control group in terms of mortality rate. Fractions 5 to 8 caused the highest mortalities (>90%) (Figure 3A). After a 14‐day exposure, fractions 1 to 4 caused mortality rates lower than 80%, while fractions 5 to 9 resulted in 100% larval mortality, and fractions 10, 11, and 12 caused mortality rates between 25% and 35% (Figure 3B). When evaluating larval biomass accumulation, it was observed that all fractions affected larval weight gain, differing from the control group on both the 7th day (*F*_12,26_ = 10.546; *p* < 0.001) and 14th day (*F*_7,16_ = 14.515; *p* < 0.001). Fractions 5, 6, 7, 8, and 9 were the most effective in reducing larval biomass accumulation. On the 7th day of exposure to the fraction in the diet, the surviving larvae weighed significantly less than those in the other treatment groups (Figure 4).

### 2.4 Chemical Characterization of the Ethyl Acetate Partition

**FIGURE 5:**
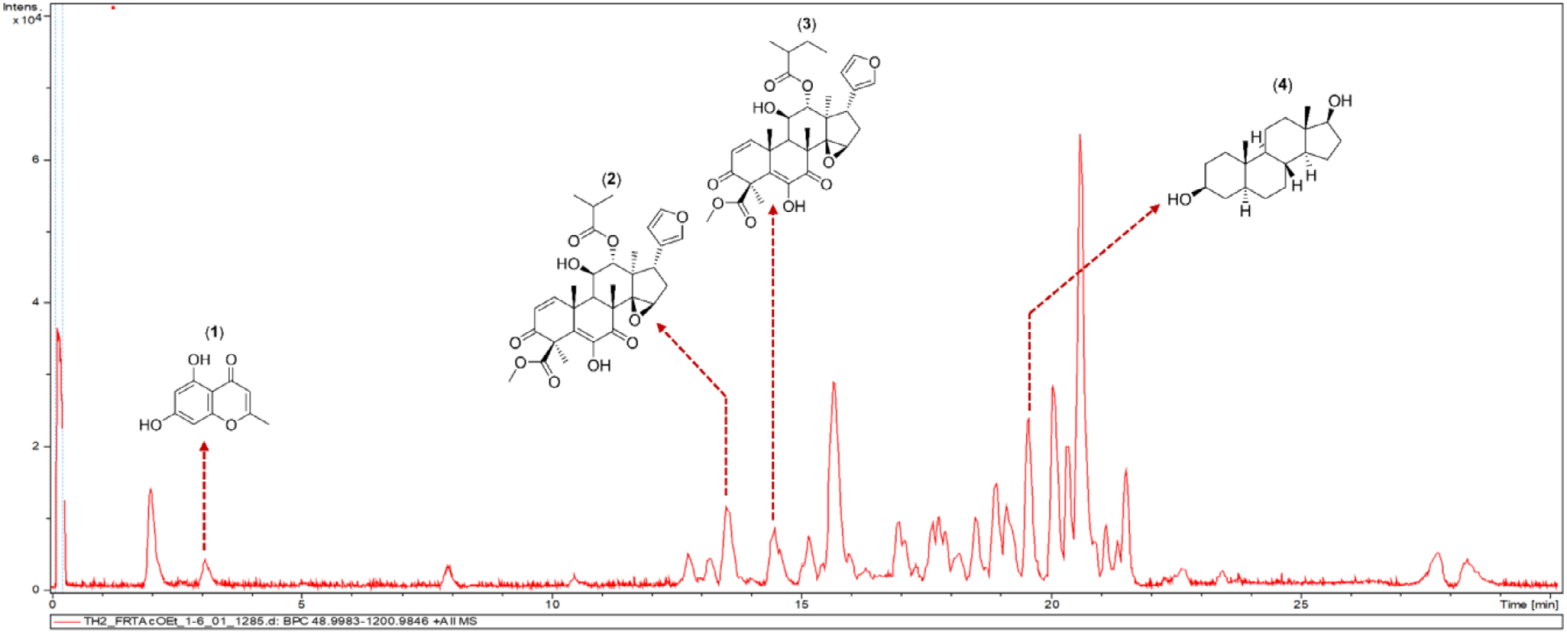
Base peak chromatogram of the ethyl acetate partition.

Sample analysis using high‐performance liquid chromatography coupled with mass spectrometry (ESI‐MS/MS) allowed the identification of 4 substances based on their fragmentation patterns. These were Noreugenin (1), with a retention time (RT) of 3.1 min; Methyl 6,11*β*‐dihydroxy‐12*α*‐(2‐methylpropanoyloxy‐3,7‐dioxo‐14*β*,15*β*‐epoxy‐1,5‐meliacadien‐29‐oate (2), RT = 13.5 min; Hirtinol A (3), RT = 14.5 min; and 5*α*‐Androstane‐3*β*,17*β*‐diol (4) RT = 19.6 min (Figure 5).

The mass spectrum obtained for compound 1 showed a fragment corresponding to the protonated molecular ion [M + H]^+^ with *m/z* 193.0503, confirming the proposed structure (Scheme S1). Compound 2 exhibited a mass spectrum with characteristic fragments of the limonoid methyl 6,11*β*‐dihydroxy‐12*α*‐(2‐methylpropanoyloxy)‐3,7‐dioxo‐14*β*,15*β*‐epoxy‐1,5‐meliacadien‐29‐oate, with *m/z* 569.2355 [M + H]+ and *m/z* 591.2166 [M + Na]^+^ [55]. The mass spectrum obtained for compound 3 showed fragments at *m/z* 583.2461 [M + H]^+^ and *m/z* 605.2309 [M + Na]^+^. When compared to the mass spectrum of compound 2, the presence of an additional 14 Da was observed, indicating the presence of one more methyl groups in the structure (Schemes S2 and S3). The other fragments in the mass spectrum of compound 3 suggest its identification as Hirtinol A, previously identified from this species (Robaina et al., 2025). Compound 4 showed peaks at *m/z* 293.2459 [M + H]+ and 315.2300 [M+Na]+ (Scheme S3), which were consistent with the steroid androstane‐3*β*, 17*β*‐diol. The fragmentation pattern for the proposed substance, based on the mass spectrum obtained, is shown in the supplementary material (Scheme S4).

## 3 Discussion

*Trichilia hirta* is a promising plant species when considering insecticidal activity due to the production of compounds belonging to the limonoid class [22, 40]. However, the insecticidal activity of compounds produced by *T. hirta* remains poorly explored. A bioassay‐guided study was conducted to investigate which fraction of *T. hirta* crude extract exhibited the highest biological activity, since the identification of secondary metabolites in plants of the Meliaceae family is important for application in pest control [4, 41].

In the first stage of the experiments, bioassays were performed using a crude methanolic extract of *T. hirta* to evaluate insecticidal activity against *S. frugiperda* larvae. Larval mortality increased with exposure time to the extracts, and on the 14th day, 100% mortality was observed in larvae exposed to *T. hirta* crude extracts. A review published in 2018 [1] listed several crude plant extracts and their respective effects against insect species of the genus *Spodoptera*, such as ovicidal, larvicidal, growth‐inhibitory, and antifeedant effects. Among the extracts reported, the crude methanolic extract of *Azadirachta indica* A. Juss (leaves and seeds), which belongs to the same botanical family as *T. hirta* (Meliaceae), was evaluated against *Spodoptera litura*. In that study, neonate larvae originating from eggs of *S. litura* immersed in *A. indica* seed extract showed 100% mortality. Although the exposure route to the extract in the present study was oral, it is important to highlight a parallel with the results reported in the aforementioned study [42]. The results observed in the literature and in the bioassays conducted in this study suggested that the biological effect against insects may be related to the presence of secondary metabolites, such as limonoids, which are characteristic compounds of plants from the Meliaceae family [12].

The second stage of the bioassays identified the fractions with the highest biological activity. Based on the results obtained from bioassays of the ethyl acetate, dichloromethane, and butanol partitions, the ethyl acetate fraction was selected for the identification of bioactive compounds, as it exhibited higher larval mortality and a greater reduction in larval weight when compared to the other partitions.

In addition to causing high larval mortality, the ethyl acetate fraction also resulted in the greatest reduction in larval weight on the 14th day, together with the dichloromethane fraction. Similar results in terms of weight reduction and mortality may be explained by the fact that both fractions have comparable polarities, classified as medium polarity. These fractions may contain substances such as limonoids, which typically present medium‐polarity structures and biological effects that are widely attributed to plants of the Meliaceae family. The literature provides extensive evidence of lethal and sublethal effects on insects [29, 43, 44].

The ethyl acetate partition was subjected to classical column chromatography, yielding 12 fractions. All of the fractions reduced larval biomass accumulation; however, fractions 5 to 9 exhibited the most pronounced lethal effects and highly significant reductions in larval biomass. Therefore, fractions 5 to 9 were selected for further studies aimed at the isolation and identification of compounds.

The compounds identified from the ethyl acetate partition of the crude methanolic extract of *T. hirta* using LC–MS/MS belong to different chemical classes. Noreugenin (compound 1) belongs to the chromone class, while compounds 2 and 3 belong to the limonoid class, and compound 4 belongs to the steroid class. The insecticidal activity of these compounds against *S. frugiperda* is not yet well established. This is the first report of the identification of the compound noreugenin in a plant of the genus *Trichilia*. However, more information is available regarding the insecticidal activity of other compounds belonging to the limonoid class. For example, numerous investigations on the biological activities of azadirachtin, a limonoid first isolated from *A. indica*, have been carried out [45]. It is important to emphasize that, in the current study, larvae were fed on an artificial diet containing *T. hirta* plant extracts. Under these conditions, it is possible to compare the current study to those that previously investigated the oral toxicity of azadirachtin. It is known that lepidopterans exhibit high sensitivity to azadirachtin when compared with insects from other orders [46]. Azadirachtin causes severe damage to the midgut, including necrosis of midgut cells and a reduction or complete inhibition of midgut enzyme production [47, 48]. Azadirachtin interferes with the production of hormones responsible for insect molting [49] and also affects cells and tissues by inhibiting cell division and protein synthesis, leading to muscle weakness and paralysis [50, 51, 52].

The two limonoids identified in this study (compounds 2 and 3) possess a cedrelone‐type skeleton. Based on these findings, their insecticidal activity is probably associated with the presence of these limonoids, as observed in bioassays using aril extracts of *T. catigua*, which interfered with the life cycle of *S. frugiperda*. From this extract, three limonoids were isolated: (1) 6α‐O‐acetyl‐7‐desacetyl‐14,15‐dihydro‐15‐oxo‐nimocinol, (2) cedrelone, and (3) 6α‐O‐acetyl‐7‐desacetyl‐nimocinol, all of which possess a cedrelone‐type skeleton similar to limonoids 2 and 3 identified from the ethyl acetate fraction of *T. hirta*. These limonoids could therefore be associated with insecticidal activity. Prolongation of the larval stage and reduction in pupal weight were observed when insects were treated with hexane, CH_2_Cl_2_, and methanolic extracts of the seeds, as well as in the CH_2_Cl_2_ extract of *T. catigua* exocarp [53].

Limonoids sharing the same cedrelone‐type skeleton, as observed in compounds 2 and 3, have significant insecticidal activity. Cedrelone, for instance, inhibited the growth of different larval stages of *Peridromia saucia* and *Mamestra configurata* when administered orally, topically, or via injection [54]. Antifeedant activity was reported for the compound (methyl 6,11β‐dihydroxy‐12α‐(2‐methylpropanoyloxy)‐3,7‐dioxo‐14β,15β‐epoxy‐1,5‐meliacadien‐29‐oate) against *Spodoptera littoralis*, *Spodoptera exigua*, *Heliothis virescens*, and *Helicoverpa armigera*. In addition, the cedrelone‐type limonoid deacetylhirta also showed antifeedant activity against *H. virescens* and *H. armigera* [55]. Collectively, these findings indicated that limonoids 2 and 3 may contribute to the biological activity observed in the ethyl acetate fraction.

Nine substances have been isolated and identified from the hexane extract of *Trichilia hirta* fruits, including a new cycloartane‐type triterpene named hirtinone, four protolimonoids, one lactone, one limonoid, and one sesquiterpene [22]. These substances could have similar biological activity to that observed in the present study. As demonstrated in a previous study [56], the toxicity of five triterpenoids isolated from *Junellia aspera* (Verbenaceae) was evaluated against *Sitophilus oryzae*. That study reported acute effects of these compounds, with one of them, the triterpene ucosterol, also showing antifeedant effects against *S. oryzae*. The insecticidal effects of the protolimonoid niloticin, isolated from the hexane extract of *Limonia acidissima* L. (Rutaceae) leaves, were evaluated against different developmental stages of the mosquito *Aedes aegypti*. The analysis revealed both lethal and sublethal effects when exposing eggs, larvae, and pupae to this compound, resulting in ovicidal activity, larval mortality, and pupal deformities [57].

**FIGURE 6:**
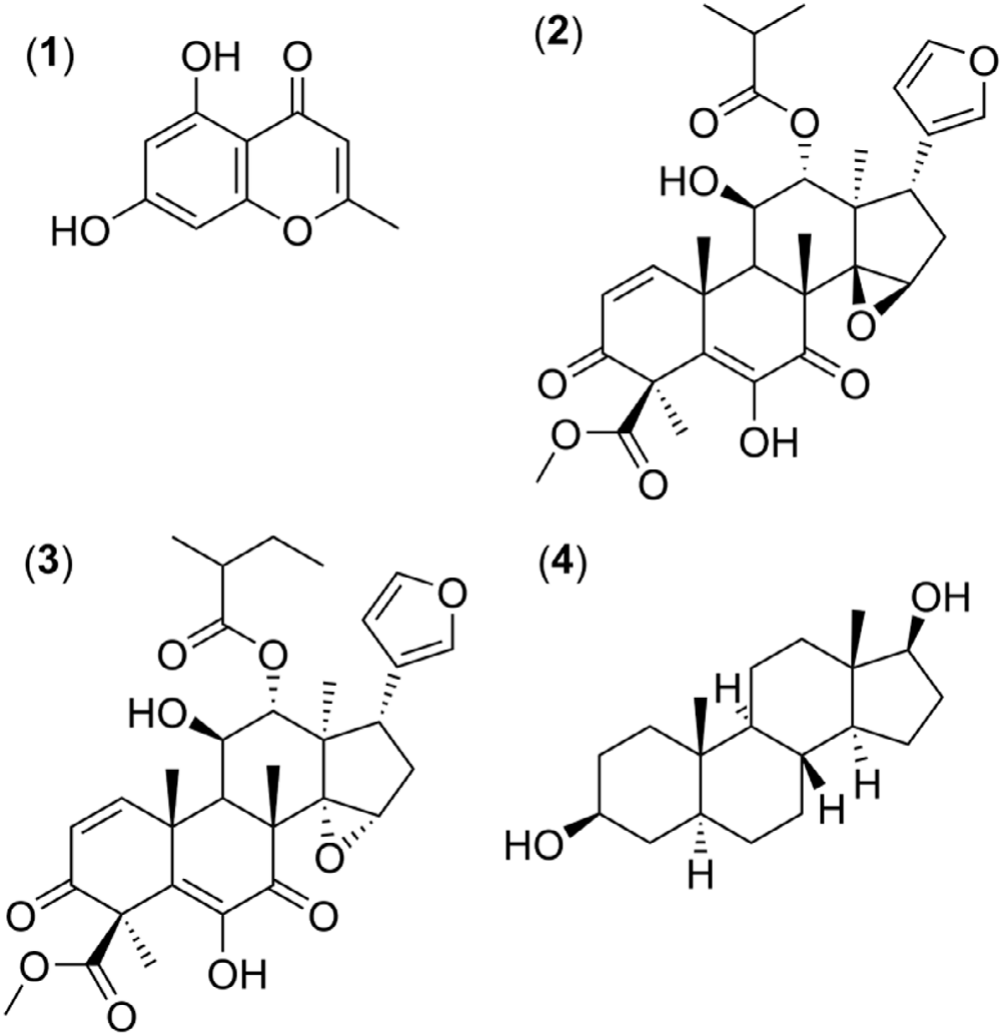
Structures of the compounds identified from high‐performance liquid chromatography coupled with ESI‐MS/MS analysis of the ethyl acetate partition.

Compounds belonging to the steroid class, androstanes and pregnanes, have been investigated, and the compound androst‐5‐en‐3β,17β‐diol 17‐acetate (an androstane) caused 100% mortality in larvae of the Colorado potato beetle *Leptinotarsa decemlineata* Say (Coleoptera) [58]. This compound has a structure similar to androstane‐3β,17β‐diol, which was identified in the present study (Figure 6). As for chromones, such as noreugenin, there are currently no reports of insecticidal activity.

This is the first study to report lethal and sublethal effects (weight reduction) of *T. hirta* extracts on *S. frugiperda*. Sublethal effects occur when substances present in plants alter insect metabolism, growth, weight gain, longevity, and fecundity [24]. Plants of the Meliaceae family are known to produce limonoids, which are triterpenoid compounds with well‐documented insecticidal activity against various insect species [52, 53, 54, 55, 56, 57, 58, 59, 60]. In addition to limonoids, other groups of compounds may have contributed to the insecticidal effects of *T. hirta* extracts. Extracts from the wood and bark of *T. hirta* and *T. glaba* inhibited growth and feeding of *Peridroma saucia* (Lepidoptera: Noctuidae) and larvae of *S. litura*, although metabolic analyses did not detect limonoids in these extracts [30]. According to the same study, plant chemical defense depends on mixtures of compounds, and crude plant extracts are often more active than isolated or purified substances [61]. The results shown here confirmed that *T. hirta* produces a range of bioactive compounds with the potential to be used as models for novel plant‐based insecticides.

## 4 Conclusions

*Trichilia hirta* methanolic crude extracts had insecticidal effects and reduced weight gain in *S. frugiperda* larvae. Among the *T. hirta* partitions tested here, the ethyl acetate partition caused the highest mortality rate and greatest reductions in biomass accumulation of *S. frugiperda* larvae. Specifically, fractions 5, 6, 7, 8, and 9 from the ethyl acetate partition were the most effective in reducing weight gain and survival of *S. frugiperda* larvae after 14 days. The chemical analysis of the ethyl acetate partition allowed the identification of 4 compounds: (1) Noreugenin, (2) methyl 6,11*β*‐dihydroxy‐12*α*‐(2‐methylpropanoyloxy‐3,7‐dioxo‐14*β*,15*β*‐epoxy‐1,5‐meliacadien‐29‐oate, (3) Hirtinol A, and (4) 5*α*‐androstane‐3*β*,17*β*‐diol. This is the first time that Noreugenin has been identified from *T. hirta*.

## 5 Experimental Section

### 5.1 Plant Material

*Trichilia hirta* fruits were collected in June 2020 from the Vale Nature Reserve, located near Linhares, Espírito Santo State, Brazil, by Domingos A. Folli. A voucher specimen (HUENF 14592) can be found in the herbarium of the State University of the North Fluminense Darcy Ribeiro. The current work was registered in the National System of Management of Genetic Resources and Associated Traditional Knowledge (SISGEN) under n° AC8E4F3.

### 5.2 Extraction and Partitioning of *Trichilia hirta* Extracts

To prepare the crude extract, 3 kg of ground material was subjected to extraction three times in methanol (9000 mL) at room temperature (for 7 days each), and the material was filtered using standard qualitative filter paper after 7 days. This procedure was repeated weekly for up to 4 weeks. Methanol was removed from the extract using a rotary evaporator (Solab Ltd., São Paulo, Brazil) at 50°C and a pressure of −600 mm Hg. Part of the crude *T. hirta* extract (230 g) was partitioned using a liquid‐liquid partitioning technique with immiscible organic solvents in increasing order of polarity: dichloromethane (CH_2_Cl_2_), ethyl acetate (AcOEt), *n*‐butanol (ButOH) and water (H_2_O). Methanolic *T. hirta* extracts were solubilized in a mixture of 125 mL of water and 25 mL of methanol. Successive extractions were then carried out with dichloromethane (125 mL), a mixture of ethyl acetate (140 mL) and water (20 mL), and *n*‐butanol (150 mL), respectively.

### 5.3 Chromatographic Separation of the Ethyl Acetate Fractions From *Trichilia hirta* Extracts

In this step, the flask containing the ethyl acetate partition (AcOEt) was left in a fume hood to allow complete evaporation of the solvent, and the resulting dry residue was subsequently weighed. Seventy‐three grams of the ethyl acetate partition (AcOEt) were subjected to classical column chromatography (CC) using silica gel 60 (0.063–0.200 mm, Merck, USA). Methanol (CH_3_OH, 99.8%), dichloromethane (CH_2_Cl_2_, 99.5%) and *n*‐butanol (C_4_H_10_O, 99.5%) supplied by Synth (São Paulo, Brazil), were used in the mobile phase. A total of 106 fractions were collected, and by using analytical thin‐layer chromatography (TLC), it was possible to identify which fractions were similar and group them together, resulting in 12 final fractions. To test the insecticidal activity of these 12 fractions, 157.0 mg per kg of diet was used, which corresponded to approximately 1/5 of the LD_50_ (785.2 mg per kg of diet). Toxicity bioassays were carried out as stated below.

### 5.4 *Spodoptera frugiperda* Rearing and Maintenance

For all bioassays, *S. frugiperda* was reared in the laboratory. *Spodoptera frugiperda* larvae and egg masses were collected in April 2021 by Mayara Barreto de Souza Arantes from maize fields of the UENF experimental unit, located at the Colégio Agrícola Antônio Sarlo, in Campos dos Goytacazes, Rio de Janeiro State, Brazil. In the laboratory (temperature: 25°C ± 2°C; RH: 60% ± 10%; and photoperiod 14hL:10hD), the larvae were individually placed in 30 mL plastic containers and fed an artificial diet specifically adapted for their development [62]. The egg masses were placed in 500 mL plastic containers, with the inner walls impregnated with diet. When the larvae reached the third instar, they were individually placed in 30 mL containers, where they remained until they pupated. The adults emerging from the pupae were transferred to cages made from PVC tubes (20 cm height × 20 cm diameter) for oviposition. The internal walls of the cages were lined with A4 paper to aid egg collection. The adults were fed on a 10% honey solution (w/v) soaked in cotton wool. The eggs were transferred to Petri dishes and remained there until hatching. Afterward, the neonates were transferred to containers containing artificial diet.

### 5.5 Bioassay‐Guided Evaluation of Insecticidal Activity

Extracts from the three different solvent partitions (dichloromethane, *n*‐butanol, and ethyl acetate) were incorporated into the diet at a concentration of 785.2 mg of each partition per kg of diet (LD_50_), and the diet was then offered to *S. frugiperda* larvae. To evaluate the bioactivity of the crude extracts from *T. hirta* fruits, 1‐day‐old *S. frugiperda* larvae were used. After incorporating the partitions, 1 g of the diet was transferred to a 30 mL plastic container, and one *S. frugiperda* larva was added to each container. On the 7th and 14th day after setting up the bioassays, larval mortality was evaluated. A total of 14 larvae per repetition and six repetitions per treatment were used, totaling 84 larvae per treatment. The control was prepared without adding the partitions, but the diet incorporated the same volume (6 mL) of solvent solution [acetone: methanol (1:1, v/v)] that was used in the solubilization of the extracts. On the 7th and 14th day after setting up the bioassays, the weight of the surviving larvae in each treatment was evaluated using an analytical balance. The methodology was adapted from previous studies [43, 63].

To evaluate the insecticidal effect of the crude methanolic extracts from *T. hirta* fruits, bioassays were conducted as follows. Preliminary experiments were carried out using crude extracts to determine a dose‐response curve, from which the LD_50_ was estimated (see statistical analysis section). This value was then used to guide the testing of fractions obtained through partitioning with butanol, ethyl acetate, and dichloromethane. Based on these experiments, the most promising fraction was selected, and its phytochemical profile was characterized using LC‐ESI‐HRMS/MS analysis. To evaluate the bioactivity of the crude extracts from *T. hirta* fruits, newly emerged *S. frugiperda* larvae (24 h old) were used. The extract was incorporated into the diet and tested at a concentration of 4000 mg of extract per kg of diet (discriminatory dose). After incorporating the extracts, 1 g of the diet was transferred to a 30 mL plastic container, and a 24‐h‐old *S. frugiperda* larva was added to each container. A total of 14 larvae per repetition and six repetitions per treatment were used, totaling 84 larvae per treatment. The controls were prepared without extract, but the diet had the same volume (6 mL) of solvent solution [acetone: methanol (1:1, v/v)] as that used in the solubilization of the extracts. On the 7th and 14th day after setting up the bioassays, the mortality of the larvae and the weight of the surviving larvae in each treatment were evaluated. As the *T. hirta* extract caused the highest mortality and reduction of weight gain of *S. frugiperda* larvae (Table 1), this extract was selected to construct the dose‐response curve and to estimate LD_50_ and LD_90_. Six concentrations were tested in the range of 200 mg to 4 g of extract per kg of diet. The methodology was the same as that stated above. On the 7th and 14th day after setting up the bioassays, larval mortality was evaluated. The LD_50_ value (785.2 mg per kg of diet) was used as a standard to compare the toxicity of different *T. hirta* solvent fractions.

**TABLE 1:** Toxic effects of *Trichilia hirta* methanolic extracts on *Spodoptera frugiperda* larvae after a 7 and 14 day exposure to extracts incorporated into the diet.

Treatments	7 days		14 days	
	Mortality (%)	Weight (mg)	Mortality (%)	Weight (mg)
*T. hirta* extract	67.85 ± 7.86*	0.13 ± 0.06*	100*	—
Control	1.78 ± 1.78	2.1 ± 0.47	1.78 ± 1.78	85.90 ± 23.50
				

*Note*: Means followed by * indicated significant differences between the larvae exposed to extracts and the controls (*p* < 0.05). The results are shown as mean % mortality and mean fresh weight (mg) (± standard error).

**TABLE 2:** Toxicity of *Trichilia hirta* fruit extract (mg kg^−1^) to *Spodoptera frugiperda* larvae.

*n* ^a^	LD_50_ (CL 95%)	LD_90_ (CL 95%)	Slope	*x* ^2^	*p*
228	785.2 (584.4–1001.6)	2594.7 (2158.5–3314.5)	0.0007	0.94	0.62

Abbreviation: CL = confidence limits.

^a^ Number of insects tested.

### 5.6 Statistical Analysis

The data for *S. frugiperda* larval mortality and weight data were subjected to analysis of variance (one‐way ANOVA), and the means were compared to the controls using the Tukey test (Minitab, LLC, version 17, State College, PA). The mortality data were corrected using Abbott's formula (1925), and the data were used to determine the dose‐response curves by Probit analysis (*p* > 0.05) (Minitab, LLC, version 17, State College, PA) [30]. The mortalities and weights of the larvae fed diets containing *n*‐butanol, dichloromethane, and ethyl acetate fractions were subjected to analysis of variance (one‐way ANOVA), and the means were compared to the mortality and weight of the larvae from the control treatment using the Tukey test (*p* < 0.05). The mortality data and the weight of the larvae fed on diets containing the fractions from the ethyl acetate partition (12 fractions) were subjected to analysis of variance (One‐way ANOVA), and the means were compared to the mortality and weight of the larvae from the control treatment using the Scott‐Knott test (*p* < 0.05) with R software (version 4.32).

### 5.7 LC‐ESI‐HRMS/MS Characterization of the Ethyl Acetate Fraction

The ethyl acetate partition was selected for chemical profile analysis due to the promising toxicity results when compared to the other partitions. A Shimadzu High‐Performance Liquid Chromatography (HPLC) system (Kyoto, Japan), coupled to a micrOTOF‐Q II (Bruker Daltonics, Billerica, MA, USA) with an electrospray ionization (ESI) source, was used to perform the ESI‐HRMS/MS analysis. The method employed the following conditions: Mobile phase (A): ultrapure Milli‐Q water (Merck Group, Darmstadt, Germany) + 0.1% formic acid (HPLC grade), mobile phase (B): methanol (HPLC grade) + 0.1% formic acid (HPLC grade), HPLC flow rate of: 0.6 mL/min, flow rate in the mass spectrometer: 0.3 mL/min, room temperature and injection volume for HPLC: 20 µL. One milligram of the sample was dissolved in 1 mL of CH_3_OH:HCOOH (0.1%), centrifuged for 10 min, and injected into the HPLC. An ultra‐high‐performance liquid chromatography system (Shimadzu, Kyoto, Japan) coupled to a microOTOFQ II (Bruker Daltonics, Billerica, MA, USA) with an electrospray ion source (ESI) was used for ESI‐HRMS/MS analysis. LC separation was performed on a Shimadzu XR‐ODS C18 75 mm × 2.1 mm, 2.1 µm analytical column (Shimadzu, Kyoto, Japan). Injections of 20 µL were made using an autosampler. The method used a gradient protocol: 0–2 min (50% B), 2–20 min (100% B), 20–23 min (100% B), 23–25 min (50% B), 25–30 min. The mass spectrometer method parameters used were electrospray ionization source in positive ion polarity; capillary voltage of 4500 V and final plate offset of −500 V; nebulizer pressure at 0.4 bar; dry gas flow rate of 4.0 L/min, and dry heater temperature at 180°C. The samples were introduced with the help of an automatic syringe pump, using approximately 2 µg/L of each sample at a constant flow of 0.3 mL/min of HPLC‐grade methanol. Collision‐induced dissociation (CID) was obtained in the automatic MS/MS mode. The spectra were obtained from the scanning range of 100–1000 *m/z* and processed using Bruker DataAnalysis 4.0 software and expressed as *m/z*.

## Author Contributions

**Mayara Barreto de Souza Arantes**: developed the hypothesis and design for this study, performed the bioactivity experiments, characterized the compounds, and prepared the manuscript. **Guilherme Ferreira Soares Passos**: and **Wanderson Rosa da Silva**: performed the bioactivity experiments. **Renata Rodrigues da Silva Robaina, Raimundo Braz Filho, José Curcino Vieira, and Lucas Silva Abreu**: characterized the compounds. **Thalya Soares Ribeiro Nogueira**: contributed to the preparation of the manuscript. **Gerson Adriano Silva**: supervised the research and contributed to all stages of the study and manuscript preparation. **Richard Ian Samuels** performed a critical review of the manuscript. All authors analyzed and discussed the results.

## Conflicts of Interest

The authors declare no conflicts of interest.

## Supporting information




**Supporting File 1**: cbdv71104‐sup‐0001‐SuppMat.docx

## Data Availability

The authors have nothing to report.
