# Supplementary material for: Insecticidal Activity of Trichilia hirta L. (Sapindales: Meliaceae) Extracts Against Spodoptera frugiperda (Lepidoptera: Noctuidae) Larvae and the Identification of Bioactive Compounds
Source: Chem Biodivers. 2026 Mar 25;23(3):e03155. doi: 10.1002/cbdv.202503155 (PMC13019008; doi:10.1002/cbdv.202503155)
Supplement: Supplementary file 1 — Supporting File 1: cbdv71104‐sup‐0001‐SuppMat.docx [file CBDV-23-e03155-s001.docx]

**Supplementary Material**


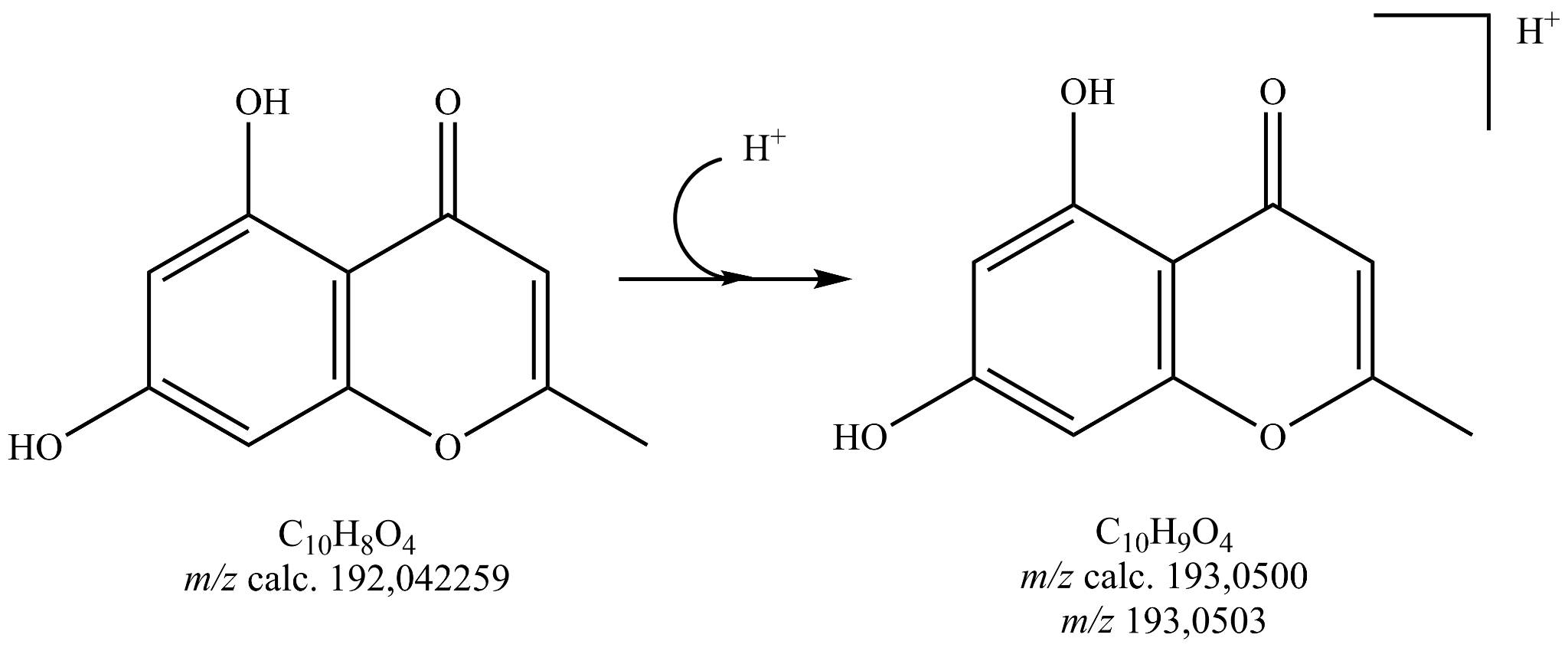


Scheme S1: Proposed fragmentation pathway of compound 1, identified with a retention time of 3.1 min.


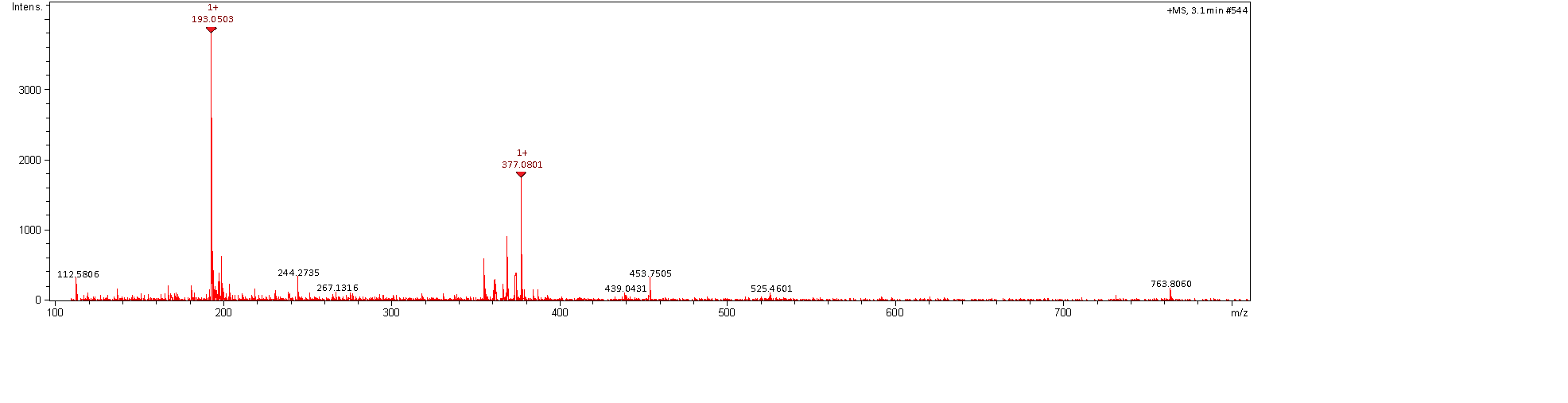


Figure S1: HR-ESI-MS (Positive ionization) spectrum of compound **1**


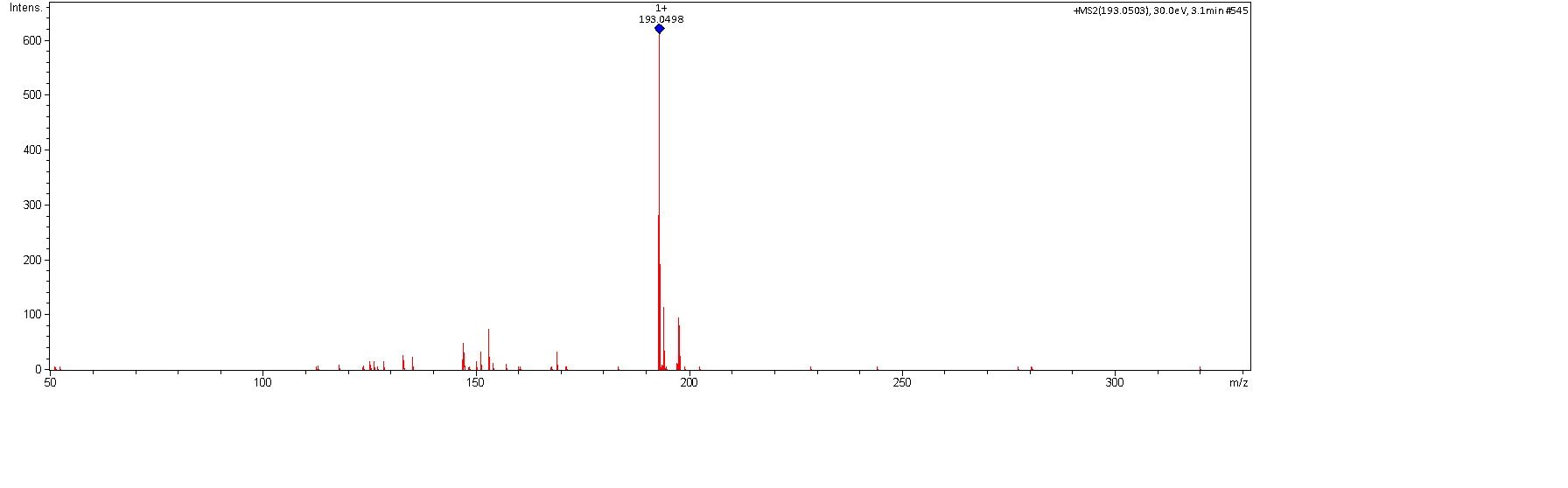


Figure S2: HR-ESI-MS (Positive ionization) spectrum of compound 1 (MS 2: m/z 193,0503)


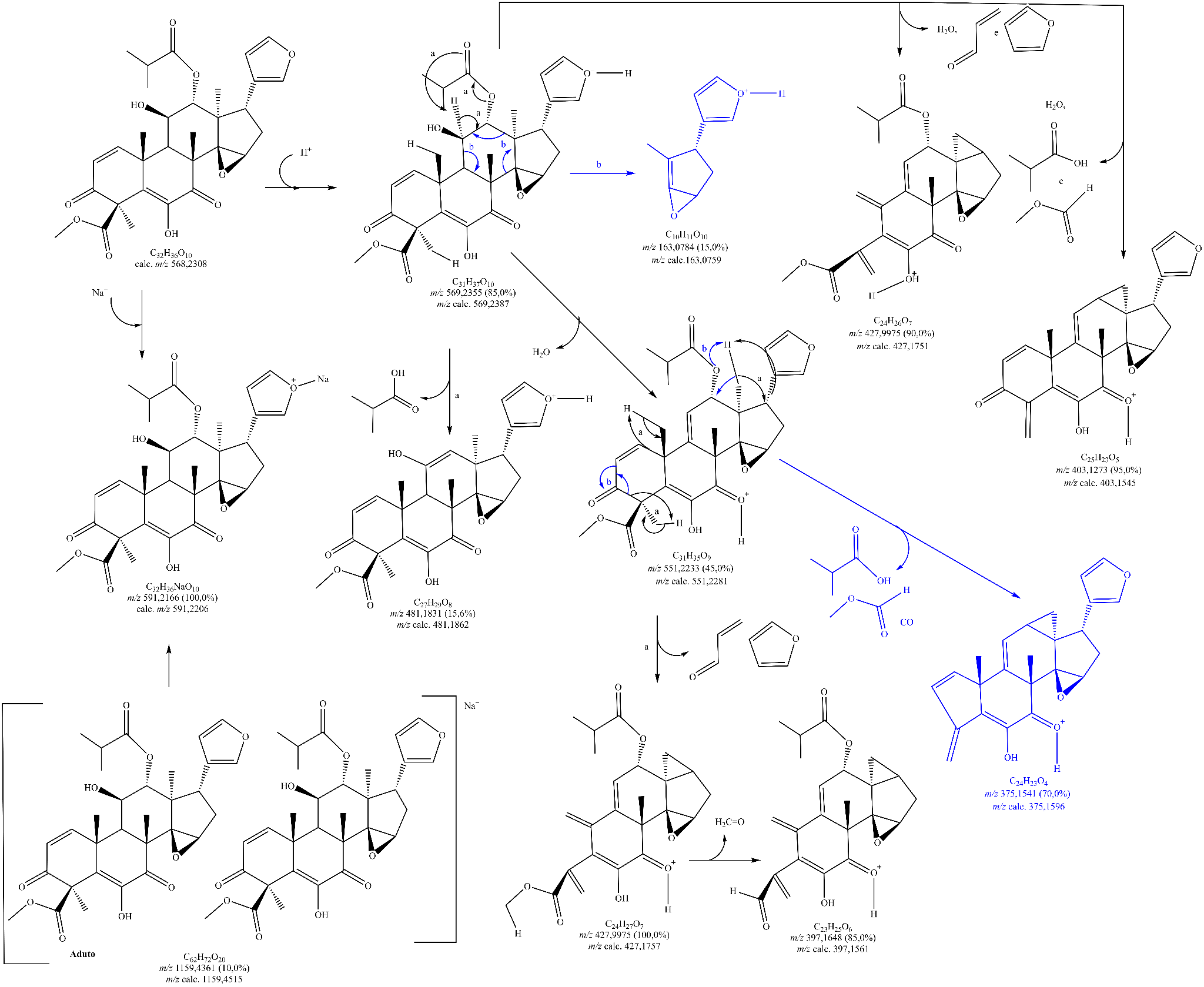


Scheme S2: Proposed fragmentation pathway of compound 2, identified with a retention time of 13.2 min.


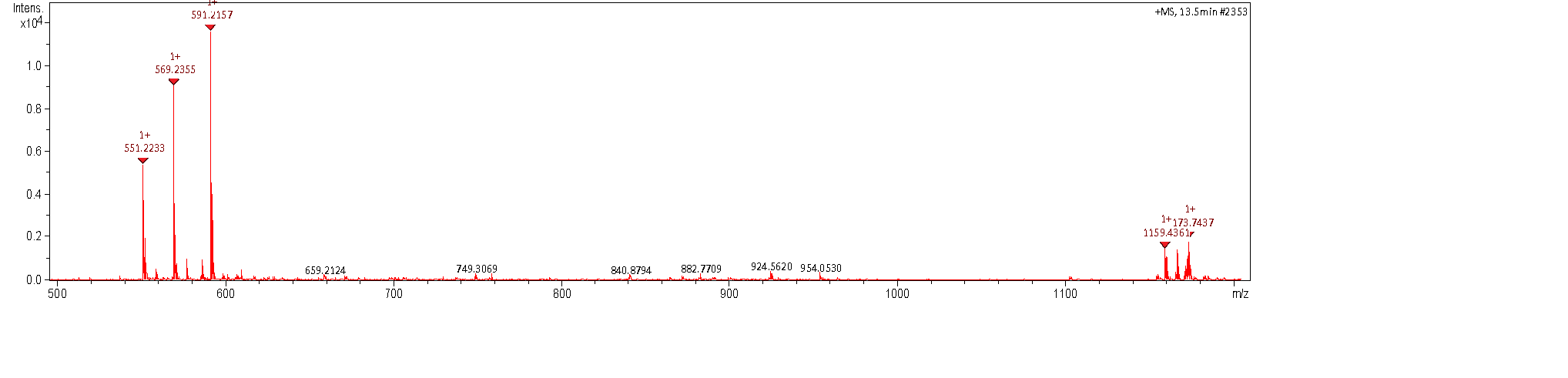


Figure S3: HR-ESI-MS (Positive ionization) spectrum of compound **2.**


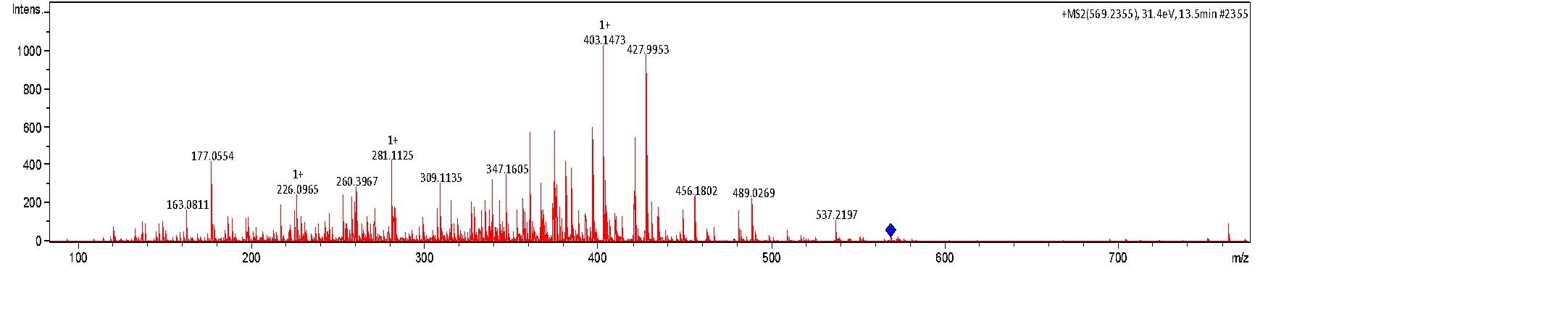


Figure S4: HR-ESI-MS (Positive ionization) spectrum of compound **2** (MS 2: m/z 569,2355)


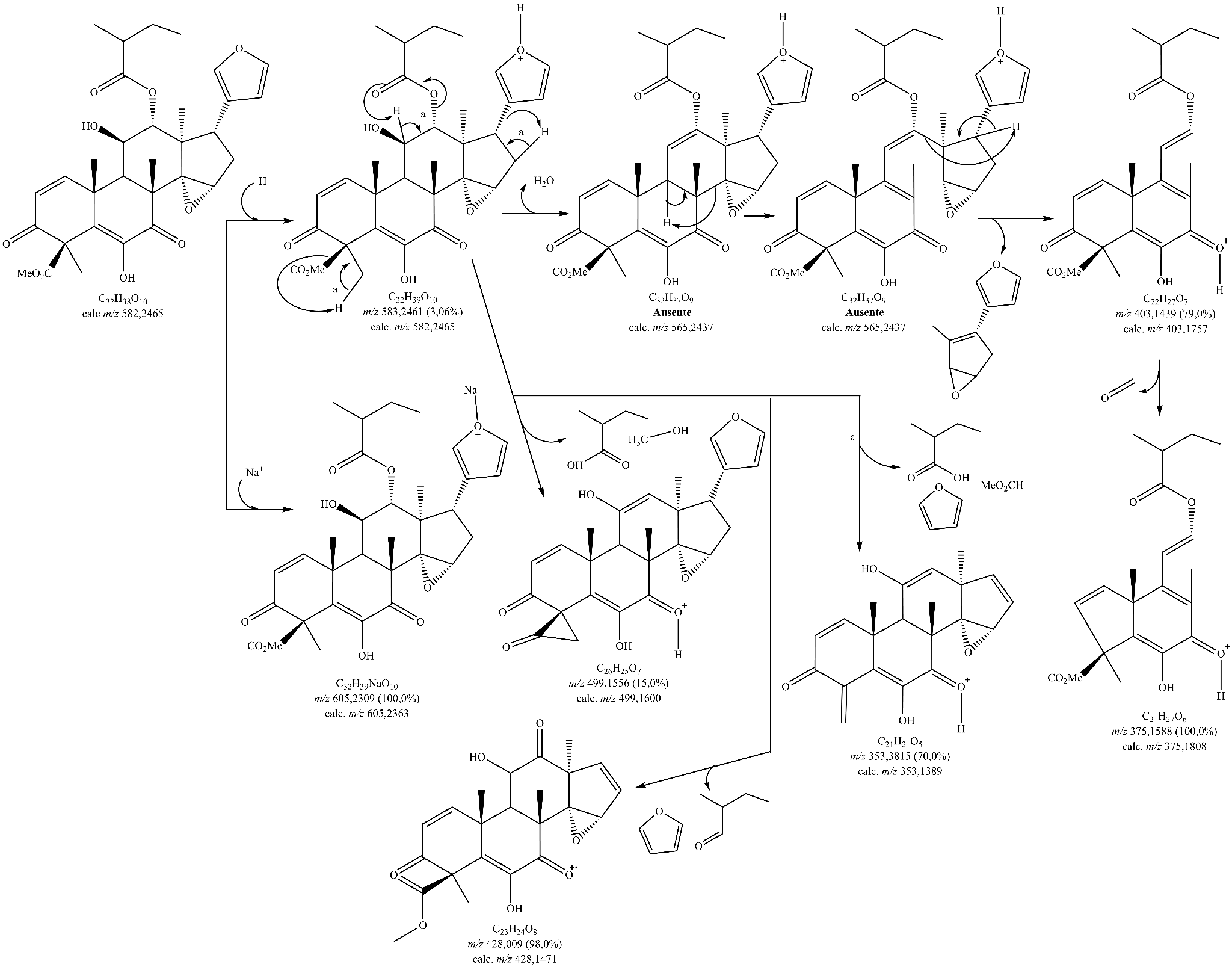


Scheme S3: Proposed fragmentation pathway of compound **3**, identified with a retention time of 14.5 min.


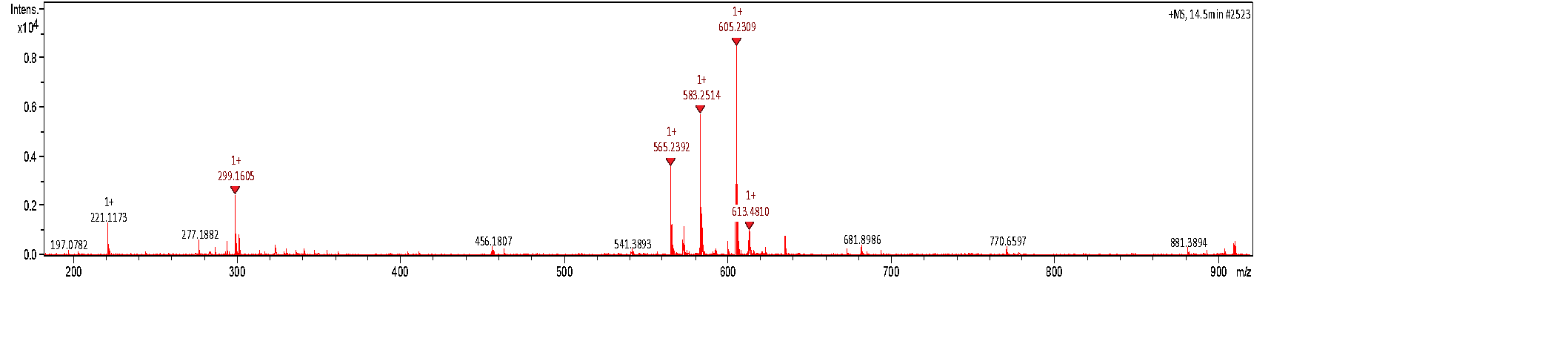


Figure S5: HR-ESI-MS (Positive ionization) spectrum of compound **3.**


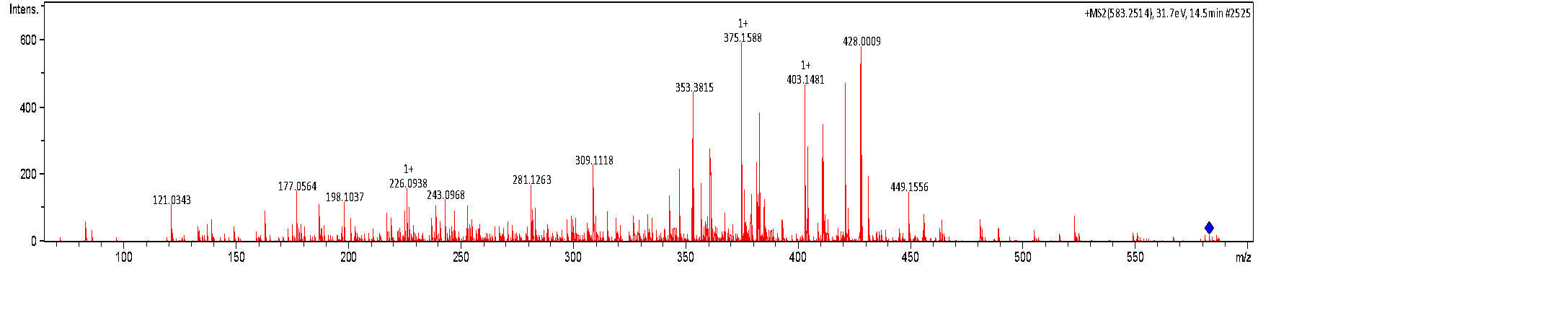


Figure S6: HR-ESI-MS (Positive ionization) spectrum of compound **3** (MS 2: m/z 583.2514)


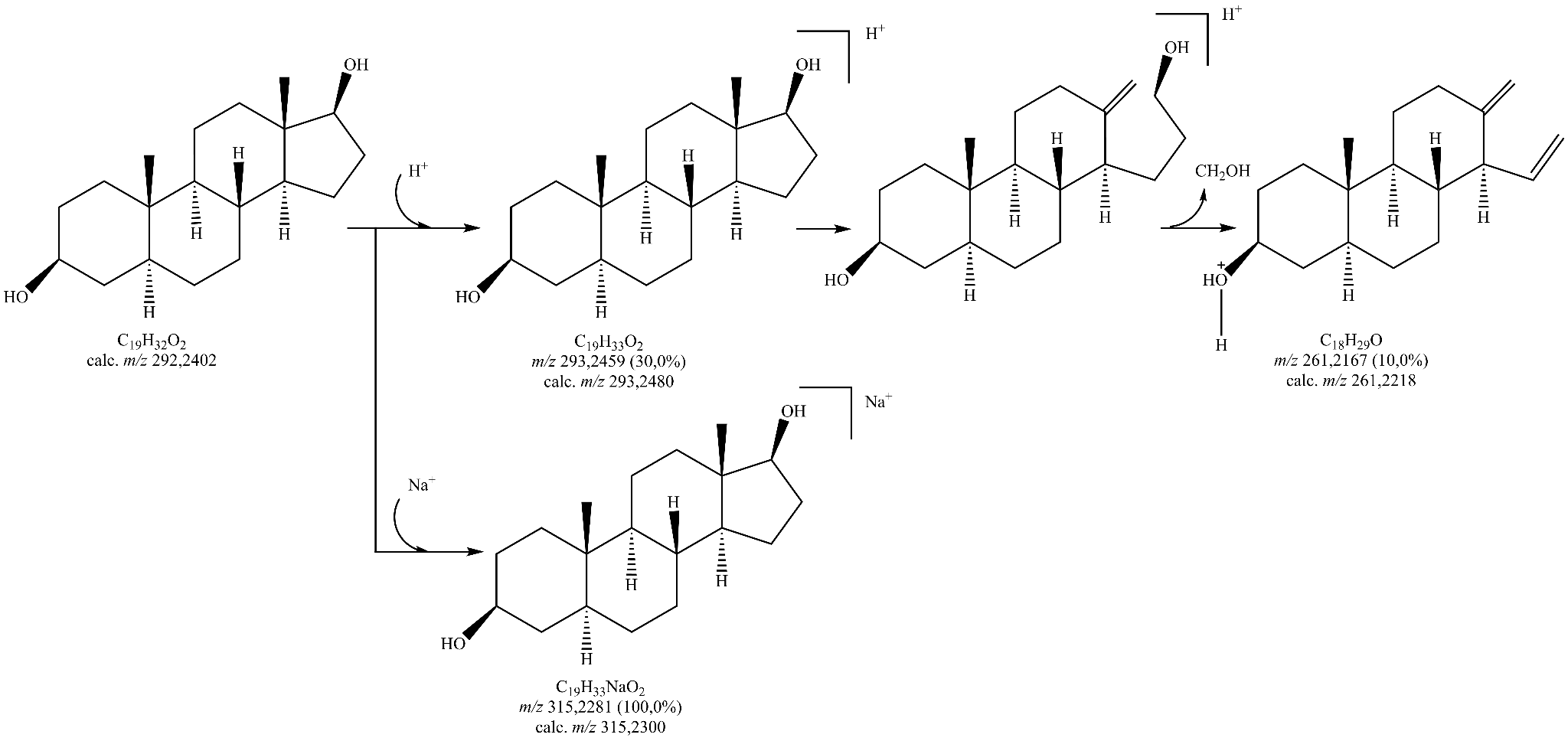


Scheme S4: Proposed fragmentation pathway of compound **4**, identified with a retention time of 19.6 min.


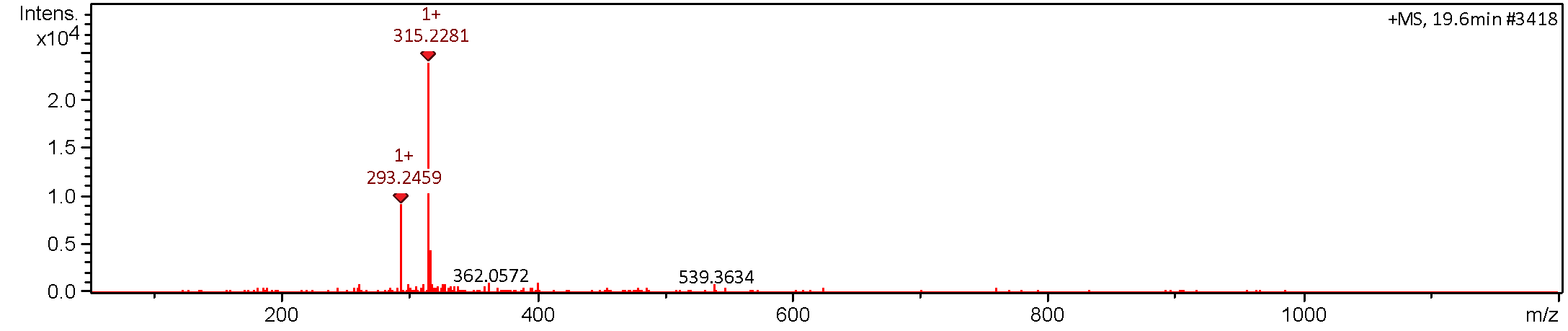


Figure S7: HR-ESI-MS (Positive ionization) spectrum of compound **4.**


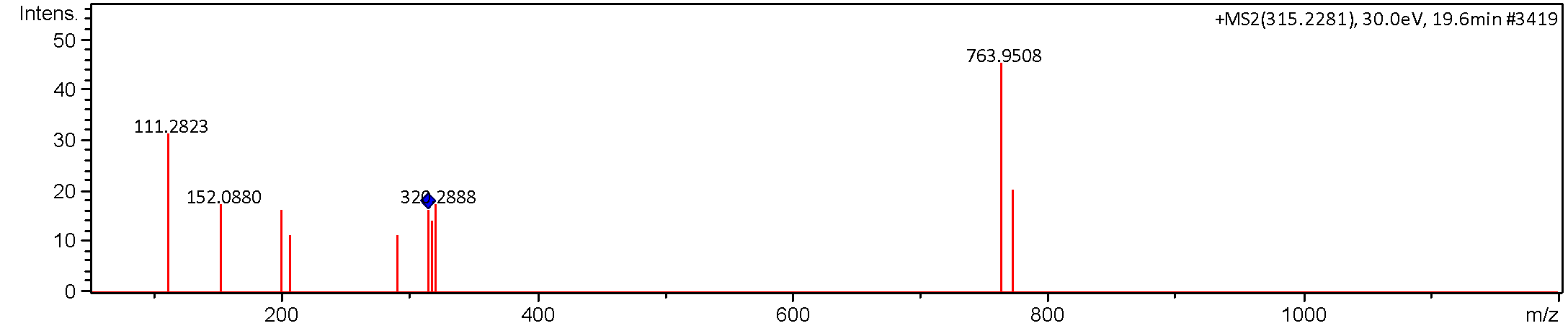


Figure S8: HR-ESI-MS (Positive ionization) spectrum of compound **3** (MS 2: m/z 315.2281)


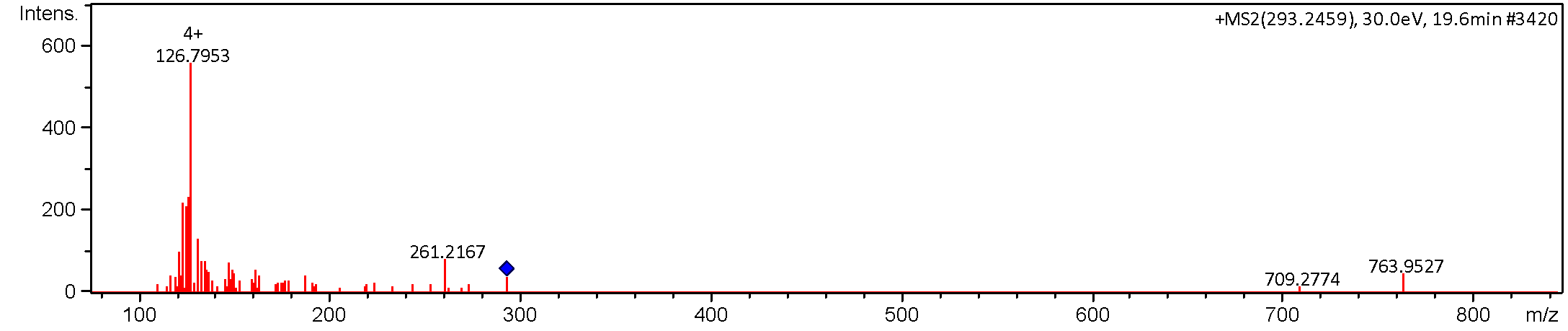


Figure S9: HR-ESI-MS (Positive ionization) spectrum of compound **3** (MS 2: m/z 293.2459)
